# Physicians’ knowledge, beliefs, and use of race and human genetic variation: new measures and insights

**DOI:** 10.1186/1472-6963-14-456

**Published:** 2014-10-02

**Authors:** Vence L Bonham, Sherrill L Sellers, Sam Woolford

**Affiliations:** Social and Behavioral Research Branch, National Human Genome Research Institute, National Institutes of Health, 31 Center Drive, Bethesda, MD 20892 USA; University Department of Family Studies & Social Work, Miami University, 210 East Spring Street, Oxford, OH 45056 USA; Department of Mathematical Sciences, Director, Center for Quantitative Analysis, Bentley University, Waltham, MA USA

**Keywords:** Scale development, Medical decision making, Personalized medicine, RACE (Racial Attributes in Clinical Evaluation), GKAI (Genetic Variation Knowledge Assessment Index), Explicit use of race

## Abstract

**Background:**

Understanding physician perspectives on the intersection of race and genomics in clinical decision making is critical as personalized medicine and genomics become more integrated in health care services. There is a paucity of literature in the United States of America (USA) and globally regarding how health care providers understand and use information about race, ethnicity and genetic variation in their clinical decision making. This paper describes the development of three scales related to addressing this gap in the literature: the Bonham and Sellers **G**enetic Variation **K**nowledge **A**ssessment **I**ndex--GKAI, **H**ealth **P**rofessionals **B**eliefs about **R**ace—HPBR, and **R**acial **A**ttributes in **C**linical **E**valuation—RACE scales.

**Methods:**

A cross-sectional, web survey of a national random sample of general internists in the USA (N = 787) was conducted. Confirmatory factor analysis was used to assess the construct validity of the scales. Scale items were developed through focus groups, cognitive interviews, expert advisory panels, and exploratory factor analysis of pilot data.

**Results:**

GKAI was measured as a count of correct answers (Mean = 3.28 SD = 1.17). HPBR yielded two domains: beliefs about race as a biological phenomenon (HPBR-BD, alpha = .69, 4 items) and beliefs about the clinical value of race and genetic variation for understanding risk for disease (HPBR-CD alpha = .61, 3 items). RACE yielded one factor (alpha = .86, 7 items).

**Conclusions:**

GKAI is a timely knowledge scale that can be used to assess health professional knowledge of race and human genetic variation. HPBR is a promising new tool for assessing health professionals’ beliefs about the role of race and its relationship with human genetic variation in clinical practice. RACE offers a valid and reliable tool for assessing explicit use of racial attributes in clinical decision making.

## Background

It is well documented that the use of race and ethnicity as surrogate markers for describing one’s risk for disease on a genomic level is common in both clinical practice [1] and research settings [2–7]. The utility of race to predict ancestry, genetic population groups and outcomes of treatment in clinical practice within the USA has been described by Barr as the “practitioner’s dilemma: can health care providers use a patient’s race to predict genetic variation, ancestry and outcomes in treatment?” [8]. We provide researchers with new survey measures to study physicians’ explicit use of patient’s race in clinical care, particularly to study health care providers’ knowledge of genetic variation, beliefs about race and genetic differences and explicit use of race in clinical decision making.

New scientific knowledge of human genetic variation is facilitating an understanding of why susceptibility to common diseases varies among individuals and populations often described by race and ethnicity [9]. Current data refute the notion that ‘races’ and ‘ethnicities’ are genetically distinct human populations as no sharp genetic boundaries can be drawn between human population groups [10]. Self-identified race and ethnicity correlate with “genetic population groups” but do not necessarily correlate with an individual’s distinct genetic background [11, 12]. Thus, there is some confusion and debate as to the relationships between risk of diseases and self-identified race [13–15].

Some researchers argue that racial and ethnic categories can serve as useful variables to investigate the genetic component of disease and patients' responses to treatment, while others contest the utility of race for understanding genetic variation [14, 16]. Many social scientists and health services researchers believe that the study of race is necessary to understand the social determinants of health [17, 18]. However, many are concerned that if physicians rely on race as a proxy for genetic risk, this could exacerbate racial and ethnic healthcare disparities in the USA and may even lead to poorer quality of care for all patients [13, 19, 20].

Some studies suggest that physicians consciously and subconsciously incorporate racial information about patients into their communication styles and decision making [21–23], but limited research has examined clinicians’ attitudes towards the relationship between race and genetic variation in clinical decision making [24]. Understanding how primary care physicians think about the intersection of race and genetics is critical for health disparities and health services research given the current racial and ethnic inequities in healthcare, the advancements of genomic medicine, and its early translation to health care.

This gap in the literature is, in part, due to a lack of reliable and valid measures to assess the role of race and genetics in clinical decision making processes. In order to assess these complex relationships, new measures, with good psychometric properties, are needed. To this end, we developed the **G**enetic Variation **K**nowledge **A**ssessment **I**ndex (GKAI) to assess physicians’ scientific knowledge of genetic variation, the **H**ealth **P**rofessionals **B**eliefs about **R**ace (HPBR) scale to measure health professionals’ beliefs about genetic difference, and the **R**acial **A**ttributes in **C**linical **E**valuation (RACE) scale to investigate health professionals’ use of race in clinical practice.^a^

In this paper, we describe the process used to develop these scales; specifically, we present a brief description of the Physicians Understanding of Human Genetic Variation (PUHGV) Study, summarize the conceptual model used to guide the process, and briefly outline the activities that generated the items used in the final scales. Finally, we describe in detail the final confirmatory phase undertaken to characterize each new measure.

### Conceptual model

**Figure 1:**
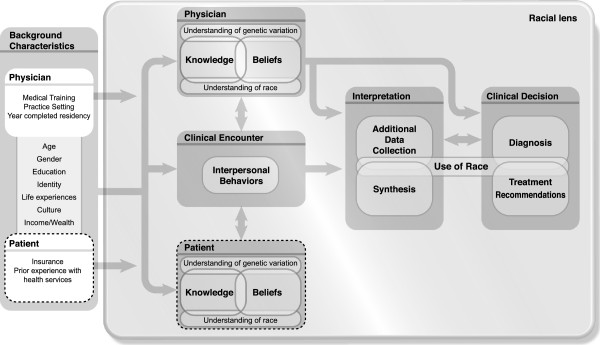
**Racial Lens In Clinical Decision Making.** The conceptual model explores the use of race and genetic variation in clinical decision making. The model consists of six domains foregrounded by a seventh domain which we describe as the racial lens. Our model suggests that this racial lens influences all aspects of the clinical decision making process.

To guide the scale development process, we developed a conceptual model aimed at exploring the use of race^b^ and genetic variation in clinical decision making (Figure 1). Our model derives from an extensive literature review and interviews with health care providers, representing an integration of critical race theory, social cognition theory, and the empirical literatures on clinical decision making, provider behavior, and the role of genetics and genomics in complex disease risk [1, 22, 25–35].

The model consists of six domains: characteristics of provider and patient; provider knowledge and beliefs; patient knowledge and beliefs^c^; the clinical encounter; interpretation; and the clinical decision. These domains foreground a seventh domain which we describe as the racial lens.

The model suggests that this racial lens is a social frame that influences the cognitive processes within all aspects of the clinical decision making process in the USA context, from the clinical encounter through synthesis of data to the final diagnostic and treatment decisions. The social frame of the racial lens permeates the clinical decision making process, is integrated both explicitly and implicitly by provider and patient, and furthers several hypotheses related to the role of race in clinical decision making. For example, it is possible that race is most influential in the interpretation and clinical decision phases, especially when genetic and molecular data are unavailable. Perhaps health professionals with greater knowledge of the science of genetic ancestry and genetic variation and its importance in diagnosis and treatment will use molecular and genomic information differently than health professionals who do not have that body of scientific knowledge [36]. Our model also suggests that genetic and molecular data could play an important role in challenging the racial lens and possibly taking the clinical encounter and thereby other domains of the clinical decision making process in directions that a racialized clinical encounter may not. It is possible that health professional’s knowledge and beliefs about race and human genetic variation could improve or could encumber their interpretation and clinical decision. We found, for example, a positive association between physicians’ anxiety due to uncertainty and self-reported use of race in their clinical decision making [37].

Thus, it is particularly important to study how physicians and other health professionals interpret and utilize race in their clinical decision making. We developed scales to measure the domains of physician knowledge and beliefs about race and human genetic variation and physician’s use of race in the domains of interpretation and clinical decision (see conceptual model). These measures were developed as part of the Physicians Understanding of Human Genetic Variation (PUHGV) Study.

The final scales presented in this report were developed within a three phased project designed to understand physicians’ knowledge of human genetic variation and the role of race in the clinical decision making process. In the first phase, focus groups with 90 general internists in the USA who self-identified as either black or white were conducted in order to gain insight into providers’ opinions on the use of race in clinical decision making and their views on the relationship between race, genetics, and disease [20, 38, 39]. Findings from these focus groups were used to develop items for the scales. We conducted 30 semi-structured cognitive interviews with general internists to assist in the development and revision of these items. Items were further refined based on guidance from two panels of experts (geneticists and survey methodologists).

In Phase II, a pilot instrument, including the three scales, was tested with a national random sample of 364 general internists. Exploratory factor analysis (EFA) was used to help diagnose areas of poor fit and to identify items with low (<.5) loadings. Items with low loadings were eliminated from the item pool and only those items with validity and strong theoretical bases were included as items in the final scales. The revised scales were then assessed by three physicians to ensure clarity and consistency among the core concepts. The current article details the methods and resulting scales from Phase III of the study.

## Methods

### Sample

The data were collected between April-December 2010; the survey was administered via the web and mail to a national sample of 2122 clinically active general internists. The sample was drawn from the SK&A company’s AMA Masterfile physician database in 2 batches. First, a general random sample (n = 1929) general internists of all racial and ethnic backgrounds was selected from the overall database. This was supplemented by a sample of physicians who graduated from historically black medical schools (n = 193) in an effort to increase the representation of black physicians in the overall sample and improve the study’s ability to detect differences between black physicians and other physicians. We excluded physicians who were not currently practicing general internists according to their office staff or did not have a current (USA) mail address, and identified a sample size of 1,738 eligible physicians.

Respondents received a $50 incentive for participating. Potential respondents received six reminders over a six month period to complete the web survey. After six contacts, non-responding physicians were sent a paper version of the survey instrument and their offices were contacted to verify the receipt of the final mailing. Of the 787 total completed surveys, 108 (13.76%) were completed and returned using the paper questionnaire. Analysis indicated no significant differences between mail and web respondents. The overall response rate for the survey was 45.35%.

This study was reviewed and approved by the National Human Genome Research Institute Institutional Review Board (05-HG-N196). The Principal Investigator requested and obtained a waiver of requiring a signed consent from each participant in the study. Participants viewed a statement that described the goals of the study, protection of personally identifiable information, and a statement that the survey was voluntary and they could withdraw at anytime. The participant checked a box that read “I agree to participate” upon checking the box the participant could begin the survey.

### Survey instrument and scale development

In Phase III, the scale items were included in a nationwide, multi-mode (web-based survey with a mail survey sent to non-responders) survey of practicing general internists concerning their knowledge and clinical application of genetics and genomics. The Health Professionals’ Genetics Education Needs Exploration (HP GENE) Survey included 81-items that included the items for three scales and 57 additional items. The final instrument consisted of six sections: (1) scientific knowledge of human genetic variation; (2) beliefs regarding genetics, race, and ethnicity; (3) consideration of race in clinical practice; (4) genetics and genomics in your clinical practice; (5) clinical decision making approaches; and, (6) demographic information. The three sections related to the scales are described below.^d^

Section 1: *Scientific knowledge of human genetic variation.* In this section (Table 1), respondents answered true or false to questions such as “the DNA sequences of two randomly selected healthy individuals of the same sex are 90-95% identical.” The items in the knowledge scale were scored true/false, with a “don’t know” category. “Don’t know” was considered an incorrect response for analytic purposes. Scores for knowledge were obtained by summing the correct responses. Higher scores indicated a greater knowledge of human genetic variation.

**Table 1:** **Items for the genetic variation knowledge assessment index (GKAI)**

ITEM#	QUESTION	^†^ANSWER
GKAI1	The DNA sequences of two randomly selected healthy individuals of the same sex are 90-95% identical.	False (22%)**
GKAI2	Most common diseases, such as diabetes and heart disease, are caused by a single gene variant.	False (80%)
GKAI3*	Common structural genetic variation (changes in the human genome such as deletions, duplications and large-scale copy-number variants) is important in health and disease.	True (90%)
GKAI4	All the genetic variation in an individual can be attributed to either spontaneous (i.e., de novo) or inherited changes in the human genome.	True (60%)
GKAI5*	The variation in the human genome includes both disease-causing gene variants and variants that have no effect on health and disease.	True (92%)
GKAI6	Individual genetic variants are usually highly predictive of the manifestation of common disease.	False (60%)
GKAI7	Prevalence of many Mendelian diseases differs by racial groups.	True (69%)
GKAI8	Self-reported race is informative of a racial group’s genetic ancestral background.	True (39%)

*Item not included in final scoring.

^**†**^Correct answer.

**Numbers in parentheses indicate the percentage of respondents who answered the question correctly.

Section 2: *Beliefs regarding genetics, race, and ethnicity.* This section comprised nine items (Table 2) used to assess physicians’ beliefs regarding the relationship between race and genetics such as “A patient’s race can identify patients who can benefit from enhanced screening for certain diseases.” The items were rated on a 5-point Likert-type scale (5 = strongly agree to 1 = strongly disagree).

**Table 2:** **Items and standardized factor loadings for the Health Professionals Beliefs about Race (HPBR) scale**

ITEM	QUESTION	LOADING
	***Biological domain***	
HPBR-BD1	Genetics usually explains differences in the prevalence of common diseases, such as diabetes and kidney disease, among racial groups.	.53
HPBR-BD2	National Census categories of race correspond with genetic differences.	.53
HPBR-BD3	Race is the best proxy clinicians have to identify genetic effects on health.	.68
HPBR-BD4	A clinician’s best predictor of treatment response is the patient’s self-identified race.	.67
HPBR-BD5*	A patient’s race provides important information about a patient’s risk of disease.	
	***Clinical domain***	
HPBR-CD1	A patient’s race can identify patients who can benefit from enhanced screening for certain diseases.	.61
HPBR-CD2	A patient’s race can identify patients who can benefit from referral to genetic services for certain diseases.	.71
HPBR-CD3	Human genetic variation provides clues to unraveling the primary causes of specific racial and ethnic disparities in health.	.47
HPBR-CD4*	There are genetic differences in racial groups that influence health.	

*Item not included in final scoring.

Biological Domain (HPBR-BD) and Clinical Domain (HPBR-CD).

Section 3: *Consideration of race in clinical practice*. This section of the survey included eight items (Table 3) to assess the degree to which health professionals employ race in their clinical decision making processes such as “I consider my patients’ race when making decisions about which medications to prescribe.” The item response categories were on a 5-point Likert-type scale (4 = all of the time to 0 = none of the time).

**Table 3:** **Items and standardized factor loadings for the Racial Attributes in Clinical Evaluation (RACE) scale**

ITEM#	QUESTION	LOADINGS
RACE1	I consider information from patients about their racial background.	.61
RACE2	I consider my patients race to better understand their genetic predispositions.	.69
RACE3	I consider my patients race when making decisions about which medications to prescribe.	.74
RACE4	I consider my patients race in determining genetic risk for common, complex diseases (e.g. kidney disease or diabetes).	.77
RACE5	I consider my patients race in making medication dosage decisions.	.64
RACE6	I consider my patients race when determining age of initiation of screening for certain diseases.	.66
RACE7	I consider my patients race in determining how aggressively to treat particular diseases.	.61
RACE8*	I consider my patients race in determining genetic risk for single gene conditions (e.g. cystic fibrosis or sickle cell disease).	

*Item not included in final scoring.

### Analysis

Descriptive statistics were calculated for the items associated with all three scales. Confirmatory factor analysis (CFA) was performed using AMOS 18 to evaluate the measurement models for the **H**ealth **P**rofessionals **B**eliefs about **R**ace (HPBR) and **R**acial **A**ttributes in **C**linical **E**valuation (RACE) scales, to identify measurement model changes and to validate each of the resulting scales. EFA was used to help diagnose areas of poor fit and to revise the scales for HPBR.

## Results

### Respondents

The demographic characteristics of the respondents (N = 787) can be found in Table 4. The sample consisted of almost twice as many males (65.3%) as females (34.7%) and 75.4% graduated from a medical school in the USA. While 88.7% of the respondents indicated that they did not receive genetics training in their primary specialty training, 84.7% indicated that their knowledge of genetics was fair or better. The majority of respondents (59.1%) practice primarily in an office setting, spend an average of 85% (SD = 19.4%) of their time seeing patients and have an average of 16.4 (SD = 9.6) years in practice post training.

**Table 4:** **Characteristics of physician respondents and U.S. internal medicine physicians**

Sample characteristics ^†^	N	%	Mean	SD	AMA* (%)
Total internal medicine physicians	787	--	--	--	
Mean age	767	--	48.6	9.6	--
Gender					
Male	505	65.3	--	--	67.2
Female	269	34.7	--	--	32.8
Ethnicity					
Hispanic/Latino	27	3.5	--	--	4.9
Race					
White	515	67.1	--	--	44
Black or African-American	49	6.4	--	--	3.9
Asian	160	20.8	--	--	17.4
American Indian/Alaska Native	9	1.2	--	--	0.1
Native Hawaiian/Pacific Islander	2	0.3	--	--	--
Other	54	7	--	--	2.3
Are you a graduate of US Medical School?					
Yes	584	75.4	--	--	--
No	190	24.5	--	--	--
Did you have genetics training in primary specialty residency?					
Yes	87	11.3	--	--	--
No	684	88.7	--	--	--
Mean years in practice post-training	769	--	16.4	9.6	--
Primary practice setting					
Academic health center	89	11.4	--	--	--
Federally Qualified Health Center	23	2.9	--	--	--
Group or staff model practice HMO	62	7.9	--	--	--
Hospital based	105	13.5	--	--	--
Office based	459	59.1	--	--	--
VA healthcare system	15	1.9	--	--	--
Other	24	3.1	--	--	--
Affiliation with academic institution?					
Yes	304	39.2	--	--	--
No	471	60.8	--	--	--
Percentage of work time seeing patients	772	85	--	--	--
How would you rate your knowledge of genetics?					
Excellent	4	0.5	--	--	--
Very good	36	4.6	--	--	--
Good	184	23.7	--	--	--
Fair	433	55.9	--	--	--
Poor	118	15.2	--	--	--

^†^Questions taken from the Health Professionals’ Genetics Education Needs Exploration (HP GENE) Survey.

*N = 160,107. Data taken from the AMA’s Physician Characteristics and Distribution in the US book, 2010 Edition.

-- Data not available.

Table 4 compares the demographics of our survey respondents with demographic data available from the AMA Physician Masterfile [40]. On those demographics that permit comparison, the survey respondents appear to reflect the profile of physicians in the AMA data except that there appears to be a higher percentage of whites in our sample than indicated in the AMA data. Noting that 27.4% of the AMA data has an unknown race, it is likely that some of these physicians are white which would then better reflect the percentage of whites in our sample.

### Scale analysis

#### **G**enetic variation **k**nowledge **a**ssessment **i**ndex (GKAI)

An initial analysis of the responses of the items in the **G**enetic Variation **K**nowledge **A**ssessment **I**ndex (GKAI) indicated that over 90% of the responses for two items (GKAI3 and GKAI5) were correct. These items were deleted from the scale as they did not sufficiently differentiate respondents (Table 1). The GKAI was created by summing the number of correct responses of the remaining items. The resulting GKAI covers the full range from zero to six, has a mean of 3.28 (SD = 1.17) and is roughly symmetric and unimodal. The GKAI has statistically significant correlation with only one item of the HPBR scale and one item of the RACE scale. In both cases, these correlations are small and could be considered random occurrences.

#### **H**ealth **p**rofessionals **b**eliefs about **r**ace (HPBR) scale

**Figure 2:**
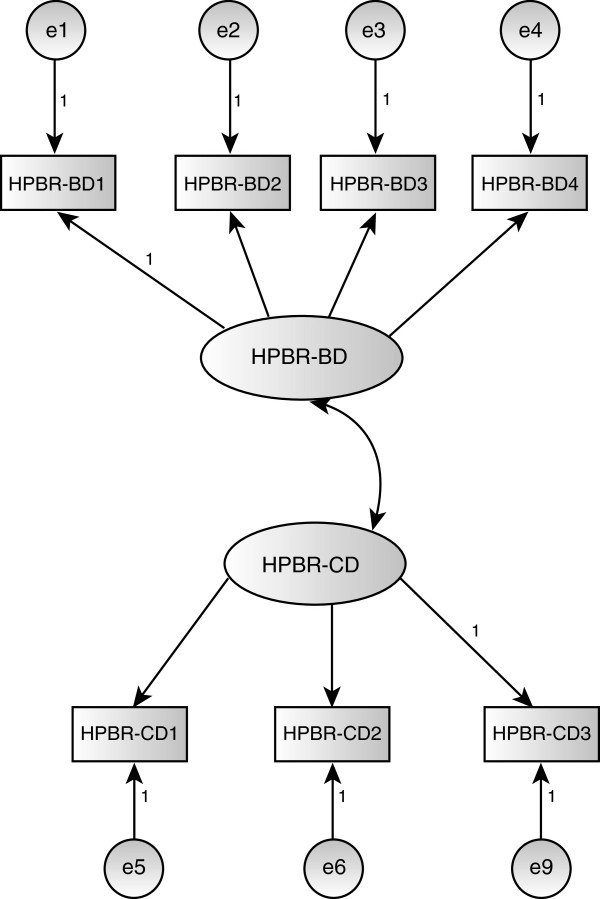
**Health Professional Beliefs and Race Scale (HPBR).** The scale consists of two domains representing beliefs about race as a biological phenomenon (HPBR-BD) and beliefs about the clinical importance of race (HPBR-CD). Confirmatory Factor Analysis for this model indicated that a correlation between the errors associated with items HPBR-BD1 and HPBR-CD3 should be included (the highest interpretable modification index).

A CFA indicated that our initial hypothesis of a single scale was not supported by the data (Table 2 & Figure 2). A subsequent EFA suggested that the HPBR scale consisted of two domains representing beliefs about race as a biological phenomenon (HPBR-BD) and beliefs about the clinical importance of race (HPBR-CD). The Cronbach’s alpha for the items included in the biological domain (HPBR-BD) was .69 and for the clinical domain (HPBR-CD) was .61, both of which are at the lower end of the range generally considered acceptable. A CFA for this model (Figure 2) was fit and the diagnostics indicated that a correlation between the errors associated with items HPBR-BD1 and HPBR-CD3 should be included. The resulting CFA was evaluated using a variety of measures [41]. The chi-square (43.6 with 12 degrees of freedom and p < .001) was significant but this could have been a result of the large sample size (n = 761).^e^ Other measures such as the GFI (.984), AGFI (.92), CFI (.96) and RMSEA (.059) all indicated an adequate fit to the data. The item loadings were all positive and statistically significant (p < .001) and the correlation was also positive and statistically significant (p < .001). The standardized loadings for HPBR-BD ranged from .53 to .68 resulting in communalities ranging from .28 to .46. The average variance extracted for HPBR-BD was .36 and the reliability was .69. The standardized loadings for HPBR-CD ranged from .47 to .71 resulting in communalities ranging from .22 to .50. The average variance extracted for HPBR-CD was .37 and the reliability was .64. The correlation between HPBR-BD and HPBR-CD is .58 and the two constructs satisfy discriminant validity. Both constructs extract less than half the variance associated with their measurement variables. This can be linked to the generally low levels of correlation (loadings) between the measurement variables and their constructs. These results suggest that the convergent validity for the constructs HPBR-BD and HPBR-CD is not as strong as typically desired. In consideration of the potential similarity between the correlated items HPBR-BD1 and HPBR-CD3, we refit the model without HPBR-BD1 and found that the fit characteristics of the resulting model along with the resulting loadings were not materially different. Consequently, we opted to present the current model.

The correlation between the HPBR-BD and HPBR-CD scales and the GKAI scale is .07 and .07 respectively. While the correlation between HPBR-BD and GKAI is just significant at the .05 level (.045) this is consistent with the large sample size and the possibly random correlations noted above.

#### **R**acial **a**ttributes in **c**linical **e**valuation (RACE) scale

**Figure 3:**
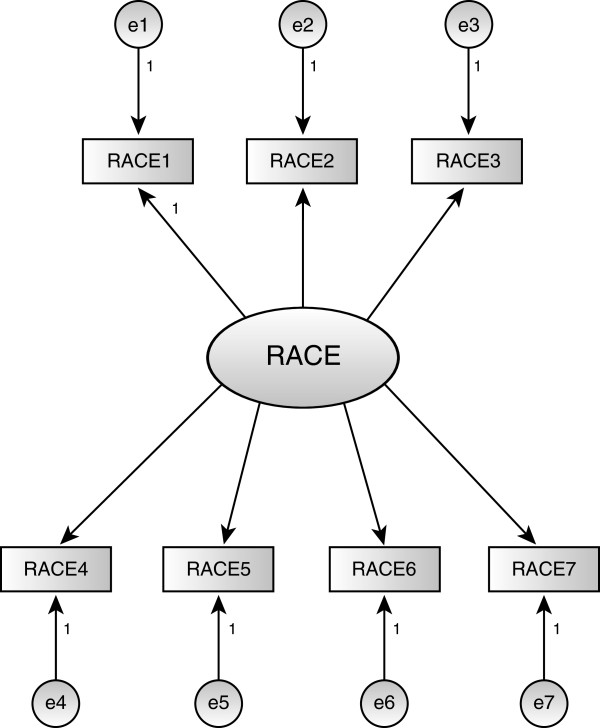
**Racial Attributes in Clinical Evaluation Scale (RACE).** The scale consists of one domain representing the explicit use of race. Confirmatory Factor Analysis for this model indicated an adequate fit to the data. There were no particularly large modification indices and no additional justifiable parameters were indicated.

Our initial hypothesis regarding the Racial Attributes in Clinical Evaluation (RACE) scale included all items indicated in Table 3. A CFA and associated diagnostics indicated that RACE8 should be dropped (Figure 3) and that correlations between the errors associated with items RACE1 and RACE2 and between the errors associated with items RACE5 and RACE7 should be added, which we did to be consistent with the theoretical intent of the scale. Incorporating these changes, Cronbach’s alpha for the remaining items used to measure the RACE scale is .86. The subsequent CFA model fit was evaluated using a variety of measures. The chi-square (46.4 with 12 degrees of freedom and p < .001) was significant but this could have been a result of the large sample size (n = 761). Other measures such as the GFI (.984), AGFI (.963), CFI (.984) and RMSEA (.061) all indicated an adequate fit to the data. The final loadings were all positive and statistically significant (p < .001) and the error correlations (ρ(RACE1, RACE2) = .424, ρ(RACE5, RACE7) = .174) were also positive and statistically significant (p < .001). The standardized loadings ranged from .61 to .77 resulting in communalities ranging from .38 to .59. The average variance extracted was .46 and the reliability was .85. These results support convergent validity of the construct [41]. The correlation of .07 between RACE and GKAI is insignificant (p > .05) while there is a significant correlation of .51 and .50 with HPBR-BD and HPBR-CD respectively.

## Discussion

We developed three measures that assess physicians’ knowledge and beliefs about genetics and genetic variation and the use of race in clinical decision making. These scales were the outgrowth of the PUHGV study qualitative research phase [20, 38, 39] and a conceptual model which aimed to facilitate exploration about how physicians’ understanding of genetic variation and race might influence the clinical process (Figure 1). We believe that the measures have the potential to lead to new insights into the intersections of race, genetics, and health.

The Genetic Variation Knowledge Assessment Index (GKAI) measures health professionals’ knowledge of human genetic variation, in contrast to health professionals’ perceived knowledge (in analysis not shown, the correlation between GKAI and physicians’ self-reported rating of their knowledge of genetics was .16). The GKAI was developed to measure specific domains of factual knowledge including concepts that are deemed important by professional organizations [42]. The GKAI could be administered as a pre-post assessment of health professional knowledge of race and genetic variation.

In contrast to measuring scientific knowledge, the beliefs scales aimed to tap into often implicit assumptions about race, although these assumptions may be drawn from experiential knowledge. The belief scales aim to capture an important, though somewhat understudied and under theorized area of medical decision making. Analysis of items to measure beliefs about race suggested two domains, one related to beliefs about race as a biological phenomenon (HPBR-BD) and the other related to beliefs about the clinical value of race and genetic variation for understanding individual and group-level risk for disease (HPBR-CD); neither scale exhibited strong psychometric properties. It is possible that there are too few items to adequately measure the constructs. Nonetheless, the HPBR is an important step in developing a measure that captures health professionals’ beliefs about the role of race in clinical practice. With further development, the HPBR may be useful for both designing interventions and in evaluating them. The scale could be used in clinical settings to aid health care providers in more explicitly articulating their beliefs about race. A clearer articulation of racial beliefs could help health care professionals better tailor care plans to individual patients [43] and communicate the complex concept of genetic variation to the general public. Future research would also do well to more explicitly link beliefs about race with knowledge about racial inequality and social causes of health disparities.

The RACE scale, developed to assess the use of race in clinical practice, had good psychometric properties. Having a valid and reliable measure of physicians’ use of race has a number of important applications. For example, the RACE scale could be utilized in cultural competency curricula to explore the use of race in clinical care. With the goal of reducing racial and ethnic health disparities in recent years, there has been an increase in cultural competency training in the health professions. Although varied in curricular and content areas, these interventions target some aspects of knowledge, attitudes/beliefs, and behaviors of health care professionals [44]. Cultural competency training seeks to impact knowledge, skills and behaviors of health care professionals, but few interventions use objective assessments to measure changes in behavior [44]. The RACE scale measures self-reported behaviors and to our knowledge is the first measure of its kind. The RACE scale intentionally does not provide the respondent a definition of race. The measure was developed for the survey respondent to answer the items based upon their own interpretation of “race” and how they use it in clinical practice. The scale is a useful tool for assessing health care provider decision making and as a tool for use in health professionals’ education.

More broadly, the appropriate use of race in clinical decision making remains contested [8] and the RACE scale aims to provide empirical data to address this debate. On one hand, use of race may heighten physicians’ recognition of social and cultural experiences of the patient and assist with clinical diagnoses based upon varying prevalence of diseases and conditions. On the other, using race may preclude personalized medicine and have life-threatening implications for failure to diagnose a disease. The value of the RACE scale is that it has the potential to measure the use of race in clinical decision making.

### Limitations

This study has four important limitations that merit discussion and further research. These limitations are related to the sample, survey response rate, the sociopolitical context in which race, genetics and health are embedded, and the exploratory nature of the study. The sample was limited to primary care physicians in the USA. General internists were selected because they are on the frontlines of health care delivery and many treat adult patients with diverse backgrounds, health concerns and risk profiles. Nonetheless, the sample is not representative of all physicians. Differences in specialty, discipline, types of responsibilities, even practice volume might affect attitudes and beliefs. Nor does the sample include other health care professionals (e.g., nurses) who are also on the frontlines of healthcare services to diverse populations. For these reasons, the findings from this study cannot be generalized. Researchers who wish to use the new measures to study health professionals globally will need to establish reliability and validity for those study populations.

A number of strategies were used to maximize survey returns [45]; nonetheless the response rate was 45%, a rate not uncommon in physician surveys. Although limitations related to nonresponse are recognized in this study, the similarity of the demographics between the sample and the AMA database of physicians (see Table 4) provides some evidence that our sample is representative.

In the USA, discourse about race in general and race and genetics in particular can be controversial [38]. Respondents may have been uncomfortable with some of the survey items resulting in non-response or socially desirable responses. We excluded only 26 cases with incomplete responses on the scale items and, as far as we could determine, these appeared to be responses missing at random. Further, we aimed to minimize the likelihood of socially desirable response bias through use of web, careful consideration of question order and wording, and use of expert panels, focus groups, and cognitive interviews.

Our findings require additional research and confirmation. While researchers can employ the RACE scale with confidence about its reliability, the HPBR scale had low convergent validity. Additional validity studies are also needed for the GKAI. Further study might examine whether physicians with more knowledge of human genetic variation hold more nuanced understanding of the role of race and ethnicity in health, and whether that understanding is associated with clinical decision making.

## Conclusions

Our measures make explicit the sometimes unarticulated assumptions about relationships between race, genetics, and health. Specifically we suggest that there is a complex association between knowledge of human genetic variation, beliefs about race and genetics, and use of race in clinical decision making processes. Developing measures that assess these three dimensions are needed if medical educators and health professionals are to move forward in efforts to understand, teach, practice, and evaluate the associations between genomics and race.

These scales provide a tool to explore how physicians think about the role of race and genomics in clinical care and the use of race in treatment decisions. The scales can be used to evaluate and assess knowledge and beliefs in medical and health professional education and continuing training. As we explore implicit attitudes of race with physicians and medical students [22, 23] we must also examine explicit use of race in clinical care and health care providers’ explicit attitudes and beliefs about the importance of race in a clinical setting.

Medical advances and social and demographic changes put pressure on the skills and knowledge of the primary care physician and other health care professionals and can challenge their ability to provide comprehensive care for patients of different social, cultural, and ancestral backgrounds. With genomic and personalized medicine we may someday move beyond race and ethnicity as a surrogate for genetic variation in health care. To move beyond race, will require health care providers to have the skills to communicate to their patients the complex concepts of population groups and human genetic variation [46]. Currently however, race and ethnicity serve as proxies for factors we are only beginning to understand about the relationships between genetic variation and health, thus in this interim, we need to deepen our understanding of health care providers understanding of race and genetic variation and use of race in their decision making. We anticipate that our measures will contribute to ongoing debates about race, racial health disparities, and genetics. The measures introduced here provide researchers an opportunity to explore explicit use of race in clinical decision making and could provide new insights into health professionals’ knowledge and beliefs about race, genetics, and health.

### Endnotes

^a^The official titles of the scales are Bonham and Sellers **G**enetic Variation **K**nowledge **A**ssessment **I**ndex (GKAI), Bonham and Sellers **H**ealth **P**rofessionals **B**eliefs about **R**ace (HPBR) scale, and the Bonham and Sellers **R**acial **A**ttributes in **C**linical **E**valuation (RACE) scale.

^b^The conceptual model and scale development focused on the concept ‘race’ and not ‘ethnicity’ because beliefs about and use of ‘race’ have an important and complex history within science and medicine in the USA.

^c^The patient has an important role in clinical decisions. The focus of the current paper is on the role of the provider. Future work will consider patient domains.

^d^Future research will use the remaining sections to validate the scales.

^e^Twenty-six cases with incomplete responses on the scale items were excluded from analysis.
